# Circ_0057558 promotes nonalcoholic fatty liver disease by regulating ROCK1/AMPK signaling through targeting miR-206

**DOI:** 10.1038/s41419-021-04090-z

**Published:** 2021-08-26

**Authors:** Xi Chen, Qing-Qing Tan, Xin-Rui Tan, Shi-Jun Li, Xing-Xing Zhang

**Affiliations:** 1 Department of Pediatrics, The Second Xiangya Hospital, Central South Univeristy, Changsha, 410011 Hunan Province China; 2 Department of Biology, The Dietrich School of Arts and Sciences, University of Pittsburgh, Pittsburgh, PA 15213 USA

**Keywords:** Diseases, Gastrointestinal diseases

## Abstract

Nonalcoholic fatty liver disease (NAFLD) is one of the most prevalent chronic liver disorders that is featured by the extensive deposition of fat in the hepatocytes. Current treatments are very limited due to its unclear pathogenesis. Here, we investigated the function of circ_0057558 and miR-206 in NAFLD. High-fat diet (HFD) feeding mouse was used as an in vivo NAFLD model and long-chain-free fatty acid (FFA)-treated liver cells were used as an in vitro NAFLD model. qRT-PCR was used to measure levels of miR-206, ROCK1 mRNA, and circ_0057558, while Western blotting was employed to determine protein levels of ROCK1, p-AMPK, AMPK, and lipogenesis-related proteins. Immunohistochemistry were performed to examine ROCK1 level. Oil-Red O staining was used to assess the lipid deposition in cells. ELISA was performed to examine secreted triglyceride (TG) level. Dual-luciferase assay was used to validate interactions of miR-206/ROCK1 and circ_0057558/miR-206. RNA immunoprecipitation was employed to confirm the binding of circ_0057558 with miR-206. Circ_0057558 was elevated while miR-206 was reduced in both in vivo and in vitro NAFLD models. miR-206 directly bound with ROCK1 3’-UTR and suppressed lipogenesis and TG secretion through targeting ROCK1/AMPK signaling. Circ_0057558 directly interacted with miR-206 to disinhibit ROCK1/AMPK signaling. Knockdown of circ_0057558 or overexpression of miR-206 inhibited lipogenesis, TG secretion and expression of lipogenesis-related proteins. ROCK1 knockdown reversed the effects of circ_0057558 overexpression. Injection of miR-206 mimics significantly ameliorated NAFLD progression in vivo. Circ_0057558 acts as a miR-206 sponge to de-repress the ROCK1/AMPK signaling and facilitates lipogenesis and TG secretion, which greatly contributes to NAFLD development and progression.

## Introduction

Nonalcoholic fatty liver disease (NAFLD) is a chronic disorder with the hallmark of accumulation of excessive fat in the hepatocytes, which does not result from alcohol [1, 2]. The occurrence of NAFLD has been drastically increasing in recent years and NAFLD appears to be one of the most prevalent chronic liver disorders in the world [3, 4]. It can develop into liver fibrosis, liver cirrhosis, or end-stage liver diseases like hepatocellular carcinoma [3]. The pathogenesis of NAFLD is complex and multi-faced [5]. Currently the treatment of NAFLD is limited and mainly comprises of lifestyle intervention and pharmacological therapy [6]. However, the compliance is very poor and the drugs for NAFLD have serious side effects. Therefore, it is very necessary to understand the underlying mechanisms of NAFLD to develop novel therapeutic strategies.

Rho-kinase 1 (ROCK1) is a serine/threonine kinase expressed in many tissues, including the liver [7, 8]. It has been implicated in many cellular processes, such as cell proliferation, cell adhesion and motility, and gene expression [9, 10]. Hepatic ROCK1 level and activity have been observed higher in both the NAFLD animal and cell models, and liver-specific deletion of ROCK1 decreases lipid deposition induced by high-fat diet (HFD) [7]. It is reported that ROCK1 suppresses AMP-activated kinase (AMPK) activity, a kinase of energy sensor that modulates lipid metabolism [7, 11]. The ROCK1/AMPK pathway is essential for the lipogenesis in the liver [7]. However, what drives ROCK1 activity in NAFLD and what are the upstream regulators of ROCK1/AMPK signaling remain largely unknown.

MicroRNAs (miRNAs) are a widely studied class of small RNAs that does not code proteins but regulates gene expression of target genes by binding with their mRNAs and degrading the mRNAs or inhibiting their translations [12]. They play key roles in diverse processes including lipid metabolism [13, 14]. Many dysregulated miRNAs have been reported in NAFLD, such as miR-140 and miR-195 [15–17]. Previous studies have shown that miR-206 can reduce lipid and glucose production in human hepatocytes [18]. Further, it has been shown that miR-206 directly binds with ROCK1 in glioma cells [19]. Nevertheless, the exact role of miR-206 in NAFLD is unclear and requires further investigations.

Circular RNAs (circRNAs) comprise of a large class of noncoding RNAs that are covalently closed [20]. They were initially considered as “junk” but later studies show that they have multiple biological functions and their expressions are tissue specific and development dependent [21]. Many circRNAs have been shown to regulate gene expression by acting as microRNA sponges or protein inhibitors [20]. Dysregulated circRNAs have been implicated in many diseases, such as cancers and metabolism-related diseases [22, 23]. For instance, decreased circRNA_021412 was associated with hepatic steatosis and circRNA_021412/miR-1972 signaling was a key regulator of lipid metabolism [24]. However, the roles of circRNAs in NAFLD are yet unclear. One of the circRNAs, circ_0057558, has been shown to promote triglyceride (TG) production and the expression of circ_0057558 positively correlates with the level of TG [23]. This implies that circ_0057558 might be involved in NAFLD but the direct examination of the role of circ_0057558 in NAFLD is lacking.

Here, we fully investigated the function of circ_0057558/miR-206/ROCK1/AMPK axis in NAFLD. We found that circ_0057558 was increased while miR-206 was decreased in NAFLD using both the in vivo mouse model and in vitro cell model. miR-206 directly targeted ROCK1 and activated AMPK signaling via inhibiting ROCK1. Moreover, circ_0057558 bound with miR-206 and acted as a miR-206 sponge to suppress AMPK signaling. Knockdown circ_0057558 or overexpression of miR-206 inhibited lipogenesis and TG secretion via ROCK1. More importantly, the injection of miR-206 greatly ameliorated the accumulation of lipid in the liver induced by high-fat diet (HFD). Together, our study reveals an essential role of circ_0057558/miR-206/ROCK1/AMPK axis in NAFLD and provides novel molecular targets for the development of therapeutic strategies for NAFLD.

## Results

### miR-206 was decreased in NAFLD and overexpression of miR-206 inhibited lipogenesis and TG secretion

**Fig. 1:**
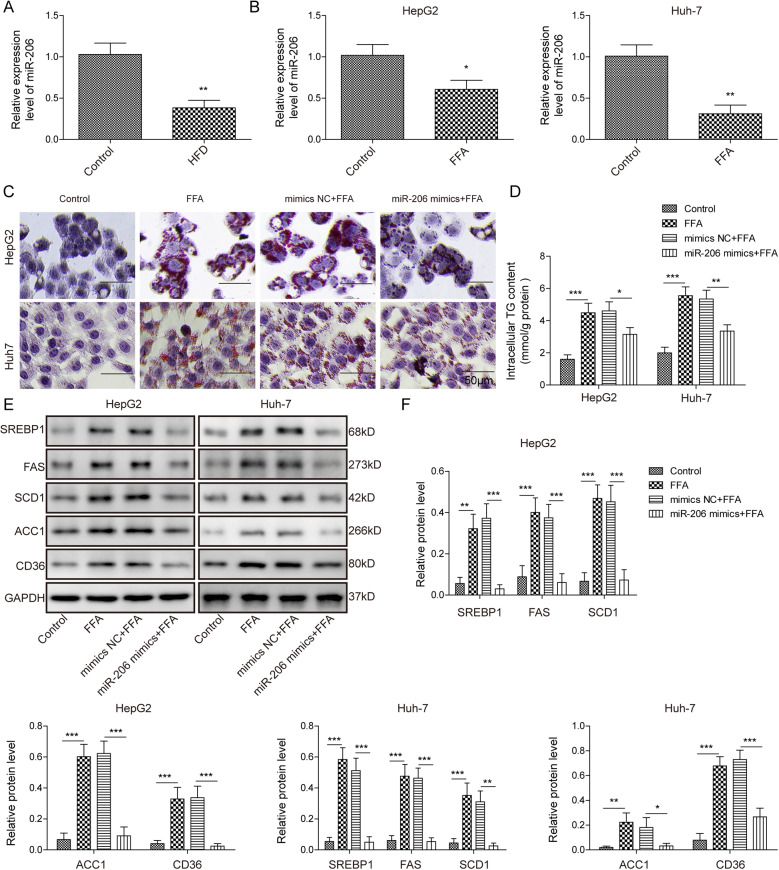
miR-206 was decreased in NAFLD and overexpression of miR-206 inhibited lipogenesis and TG secretion. **A** miR-206 level in HFD-fed mice and control mice. **B** miR-206 level in liver cells treated with FFA or control. **C** Representative oil-red o staining images of cells overexpressed with miR-206 following FFA or control treatment. **D** Secreted TG level in cells with transfection of miR-206 mimics or NC following FFA or control treatment. **E** Protein levels of lipogenesis-related proteins in cells with the overexpression of miR-206 mimics or NC following FFA or control treatment. **F** Quantifications of **E**. *p < 0.05, **p < 0.01, and ***p < 0.001.

To investigate the function of miR-206 in NAFLD, we first measured the level of miR-206 in mice with high-fat diet (HFD) induced NAFLD. Relative expression levels of miR-206 were normalized to U6 RNA. The mice were fed on HFD for 12 weeks to induce NAFLD and the liver was harvested. Compared to mice fed on a regular diet, the miR-206 level was greatly decreased in the liver of mice fed on HFD (Fig. 1A). To characterize the role of miR-206 in NAFLD, we employed the in vitro NAFLD cell model by treating human liver cells (Huh-7 and HepG2) with 1 mM long-chain FFA (oleic acid: palmitic acid = 2:1) for 1 day. Similarly, we observed a lower level of miR-206 in cells treated with FFA compared with control cells (Fig. 1B). Using oil-red o staining, we found significant lipid deposition in FFA-treated cells (Fig. 1C). TG level and lipogenesis-related proteins including SREBP1, FAS, SCD1, ACC1, and CD36 were elevated as well following FFA treatment (Fig. 1D–F). These results suggest the success of the NAFLD cell model induced by FFA treatment. Transfection of miR-206 mimics remarkably increased miR-206 level (Fig. S1A). Intriguingly, overexpression of miR-206 greatly diminished the lipid deposition induced by FFA treatment, and the TG level, as well as the expression levels of lipogenesis-related proteins (Fig. 1C–F). Together, these results show that miR-206 is reduced in NAFLD and that rescue of miR-206 level could suppress the lipogenesis process.

### miR-206 inhibited lipogenesis and TG secretion and retarded NAFLD in vivo

**Fig. 2:**
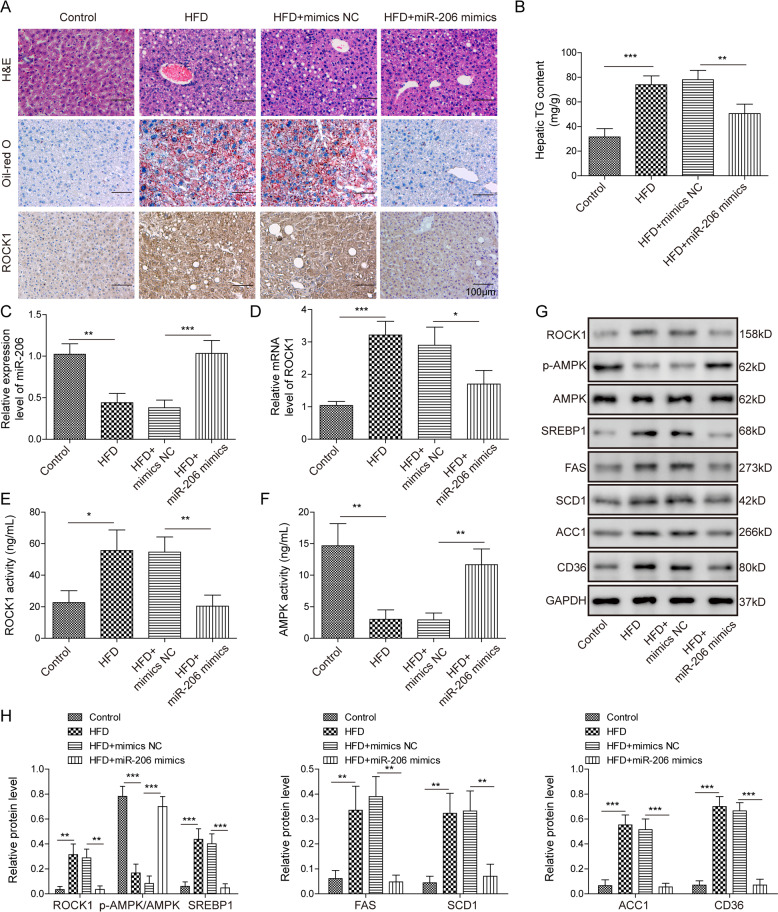
miR-206 inhibited lipogenesis and TG secretion in vivo. **A** Representative images of H&E staining (upper), oil-red o staining (middle), and IHC staining (bottom) in liver tissues from HFD-fed mice or control mice with injection of miR-206 mimics or NC. **B** Secreted TG levels in liver tissues from HFD-fed mice or control mice with injection of miR-206 mimics or NC. **C** miR-206 levels in liver tissues from HFD-fed mice or control mice with injection of miR-206 mimics or NC. **D** ROCK1 mRNA levels in liver tissues from HFD-fed mice or control mice with injection of miR-206 mimics or NC. **E**, **F** ROCK1/AMPK activities in liver tissues from HFD-fed mice or control mice with injection of miR-206 mimics or NC. **G** Protein levels of ROCK1, p-AMPK, and lipogenesis-related proteins in liver tissues from HFD-fed mice or control mice with injection of miR-206 mimics or NC. **H** Quantifications of **G**. *p < 0.05, **p < 0.01, and ***p < 0.001.

We next investigated the role of miR-206 in lipogenesis in vivo using the HFD-fed animals. As expected, HFD-induced remarkable lipid deposition in the liver and secreted TG level was elevated as well (Fig. 2A, B). Using immunohistochemistry, we also observed a higher level of ROCK1 in the liver of the HFD group compared to control mice (Fig. 2A). Also consistently, the miR-206 level was significantly diminished, while ROCK1 mRNA was increased following HFD (Fig. 2C, D). However, injection of miR-206 mimics through the vein recovered the level of miR-206 and decreased ROCK1 level (Fig. 2A, C, D). Moreover, miR-206 mimics significantly decreased the lipid deposition induced by HFD, as well as TG level (Fig. 2A, B). At the molecular level, the activity of ROCK1 was increased while AMPK activity was decreased in the liver of mice fed on HFD (Fig. 2E, F). We also found that HFD upregulated the expression levels of ROCK1 and lipogenesis-related proteins, including SREBP1, FAS, SCD1, ACC1, and CD36, but downregulated the p-AMPK level (Fig. 2G, H). Overexpression of miR-206 reversed those changes, recovering the level of p-AMPK and AMPK activity and decreased the levels of ROCK1 and lipogenesis-related proteins and ROCK1 activity (Fig. 2E–H). Taken these results together, we demonstrate that miR-206 inhibits lipogenesis and TG secretion in vivo by regulating ROCK1/AMPK signaling.

### miR-206 directly targeted ROCK1 to reduce its expression

**Fig. 3:**
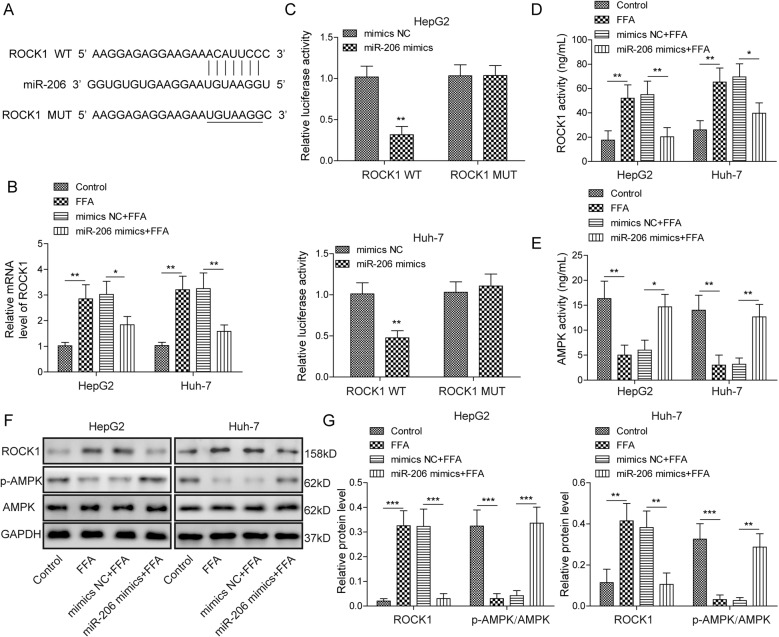
miR-206 directly targeted ROCK1 to reduce its expression. **A** Predicted binding sites between miR-206 and ROCK1 mRNA. **B** ROCK1 mRNA levels in cells with the overexpression of miR-206 following FFA or control treatment. **C** Relative luciferase activity of ROCK1-WT and ROCK1-MUT in cells with the transfection of miR-206 mimics or NC. **D**, **E** ROCK1/AMPK activities in cells with the transfection of miR-206 mimics or NC following FFA or control treatment. **F** Protein levels of ROCK1 and p-AMPK in cells with the transfection of miR-206 mimics or NC following FFA or control treatment. **G** Quantifications of **F**. *p < 0.05, **p < 0.01, and ***p < 0.001.

It is well known that miRNAs exert functions by regulating gene expressions of downstream targets [12]. Using bioinformatic analysis (Starbase, http://starbase.sysu.edu.cn/index.php), we found some complementary binding sites between miR-206 and ROCK1 mRNA (Fig. 3A). Moreover, the ROCK1 mRNA level was increased in FFA-treated cells while overexpression of miR-206 inhibited the elevation (Fig. 3B). To directly examine the interaction, we used a dual-luciferase assay. miR-206 mimics significantly diminished the relative luciferase activity of the ROCK1-WT reporter but not the ROCK1-Mut reporter wherein the binding sites with miR-206 were mutated (Fig. 3C), suggesting that miR-206 directly binds with ROCK1 3’-UTR and negatively regulates its expression. Further, as expected, FFA treatment increased the level of ROCK1 protein and its activity but decreased the level of p-AMPK1 and AMPK activity (Fig. 3D–G). Overexpression of miR-206 reversed those changes, suppressing the increase of ROCK1 level and activity and upregulating p-AMPK1 level and its activity (Fig. 3D–G). These data demonstrate that miR-206 activates AMPK signaling by suppressing ROCK1 expression.

### miR-206 inhibited lipogenesis via targeting ROCK1

**Fig. 4:**
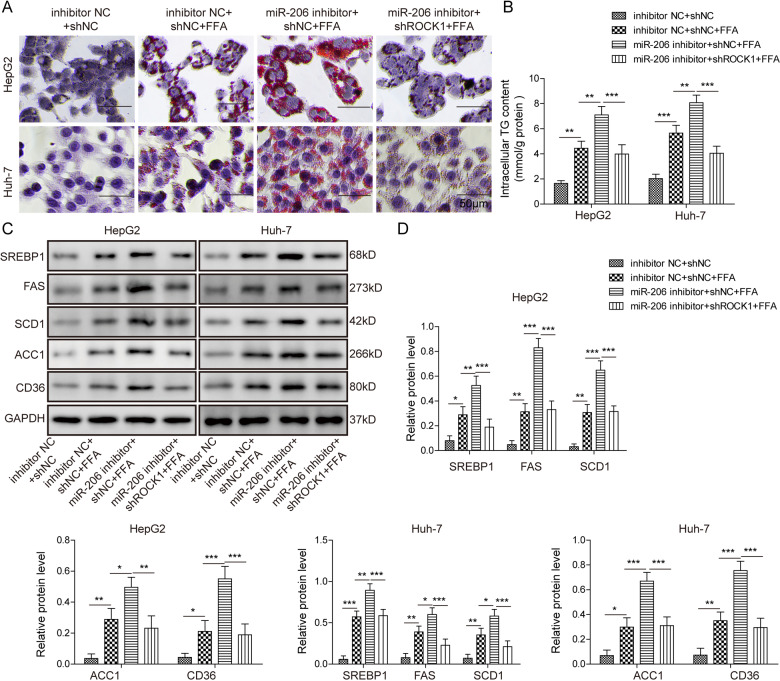
miR-206 inhibited lipogenesis via ROCK1. **A** Representative oil-red o staining images of cells with the transfection of miR-206 inhibitor or NC together with sh-ROCK1 or sh-NC following FFA or control treatment. **B** Secreted TG levels in cells transfected with miR-206 inhibitor or NC together with sh-ROCK1 or sh-NC following FFA or control treatment. **C** Protein levels of lipogenesis-related proteins in cells with the transfection of miR-206 inhibitor or NC together with sh-ROCK1 or sh-NC following FFA or control treatment. **D** Quantifications of **C**. *p < 0.05, **p < 0.01, and ***p < 0.001.

We then investigated the function of miR-206/ROCK1 interaction in NAFLD. Again, we mimicked fatty acid overload through treating liver cells with FFA, which induced lipid deposition, elevated TG secretion, and higher levels of lipogenesis-related proteins, such as SREBP1, FAS, SCD1, ACC1, and CD36 (Fig. 4A–D). Transfection of miR-206 inhibitor greatly diminished miR-206 expression in cells (Fig. S1A). Downregulation of the miR-206 level significantly promoted the effects of FFA on lipogenesis (Fig. 4A–D), suggesting that miR-206 inhibits lipogenesis. Transfection of sh-ROCK1 remarkably decreased the mRNA and protein level of ROCK1 (Fig. S1C–E). Knockdown ROCK1 drastically reversed the effects of the miR-206 inhibitor on lipogenesis (Fig. 4A–D). We, therefore, conclude that miR-206 suppresses the lipogenesis process via targeting ROCK1.

### Circ_0057558 was elevated in NAFLD and knockdown circ_0057558 inhibited lipogenesis

**Fig. 5:**
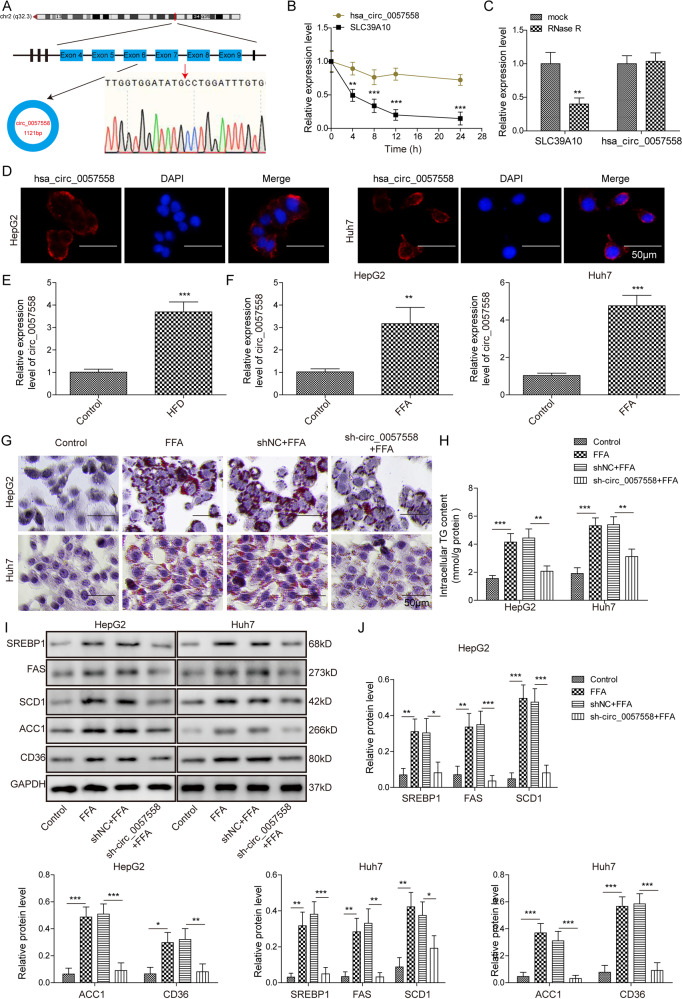
Circ_0057558 was elevated in NAFLD and knockdown circ_0057558 inhibited lipogenesis. **A** The feature of circ_0057558 and Sanger sequencing result. **B** Relative expression levels of circ_0057558 and SLC39A10 at the indicated time after antinomycin D treatment. **C** Relative expression levels of circ_0057558 and SLC39A10 after RNase R treatment. **D** FISH analysis of subcellular localization of circ_0057558. **E** circ_0057558 level in HFD-fed mice and control mice. **F** circ_0057558 level in cells treated with FFA or control. **G** Representative oil-red o staining images of cells transfected with sh-circ_0057558 or sh-NC following FFA or control treatment. **H** Secreted TG level in cells with the transfection of sh-circ_0057558 or sh-NC following FFA or control treatment. **I** Protein levels of lipogenesis-related proteins in cells transfected with sh-circ_0057558 or sh-NC following FFA or control treatment. **J** Quantifications of **I**. *p < 0.05, **p < 0.01, and ***p < 0.001.

Circ_0057558 has been implicated in TG genesis and its expression is positively correlated with cholesterol level [23]. As a relatively new circRNA, we first characterized its features. Circ_0057558 derives from exons 4–9 of the SLC39A10 gene, whose spliced mature sequence length is 1121 bp. With divergent primers and Sanger sequencing, we confirmed that the back-splice junction site of circ_0057558 (Fig. 5A). Then qRT-PCR was performed to measure the level of circ_0057558 and SLC39A10 mRNA in cultured liver cells (HepG2) treated with the transcription inhibitor Actinomycin D or processive 3’ to 5’ exoribonuclease RNase R. We found that circ_0057558 was more stable than SLC39A10 (Fig. 5B), and circ_0057558 was significantly resistant to RNase R compared with SLC39A10 (Fig. 5C). Additionally, we observed that circ_0057558 was primarily located in the cytoplasm by FISH assay (Fig. 5D). Following the characterization, we then studied its role in NAFLD. We measured its level in both the NAFLD mouse model and cell model. We found both the HFD and FFA treatment significantly enhanced circ_0057558 expression (Fig. 5E, F), implying that circ_0057558 is involved in NAFLD. Next, we determined the role of circ_0057558 in lipogenesis. Knockdown circ_0057558 through shRNA remarkably reduced circ_0057558 level (Fig. S1B), as well as the lipid deposition, TG secretion, and the expression level of lipogenesis-related proteins including SREBP1, FAS, SCD1, ACC1, and CD36 in FFA-treated cells (Fig. 5G–J). These results indicate that the knockdown of circ_0057558 inhibits the lipogenesis process.

### Circ_0057558 inhibited AMPK signaling via targeting miR-206

**Fig. 6:**
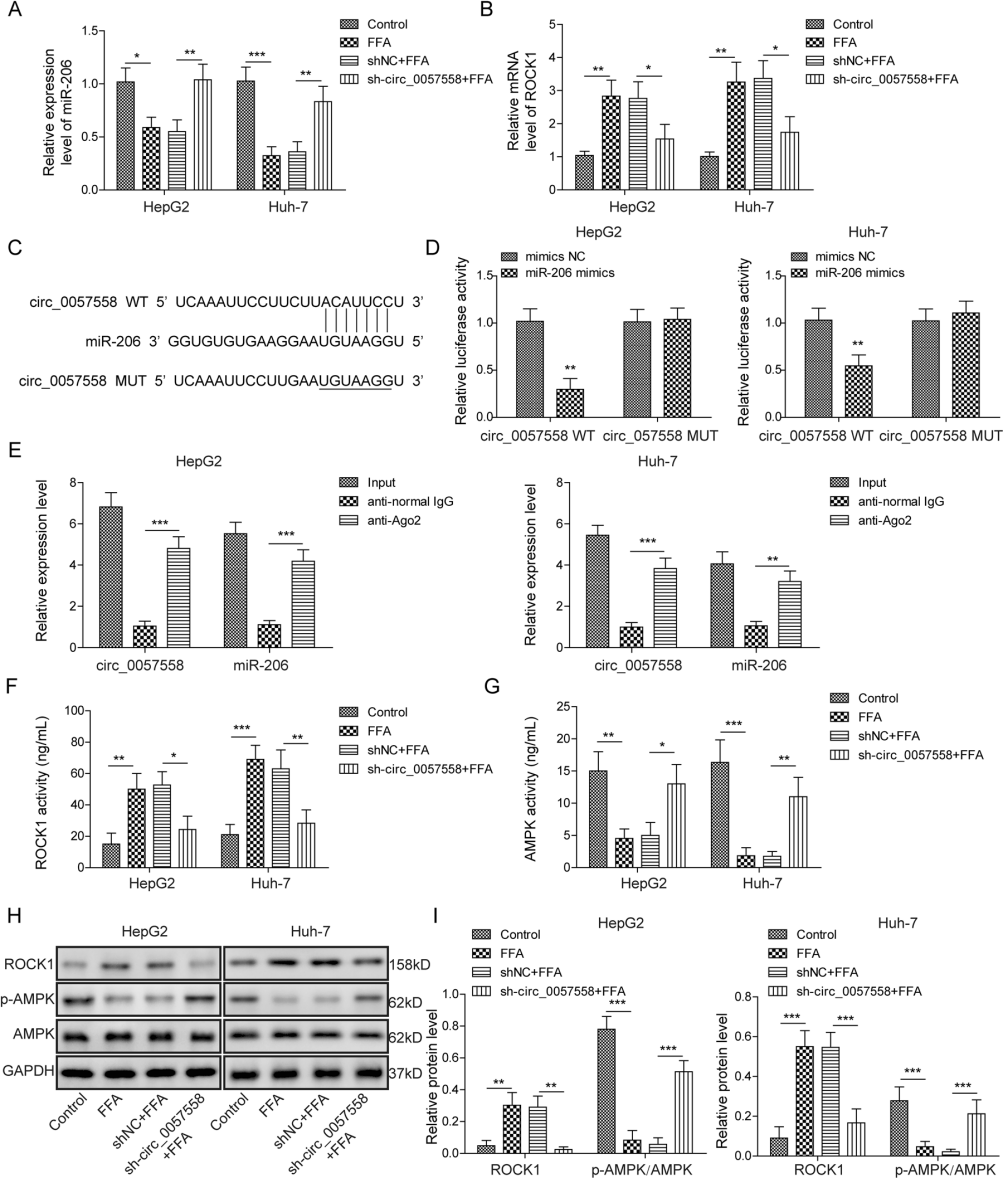
Circ_0057558 inhibited AMPK signaling via targeting miR-206. **A** miR-206 levels in cells with the transfection of sh-circ_0057558 or sh-NC following FFA or control treatment. **B** ROCK1 mRNA levels in cells with the transfection of sh-circ_0057558 or sh-NC following FFA or control treatment. **C** Binding sites between the miR-206 and circ_0057558. **D** Relative luciferase activity of circ_0057558-WT and circ_0057558-MUT in cells with the transfection of miR-206 mimics or NC. **E** circ_0057558 and miR-206 levels following immunoprecipitation with anti-AGO2 or anti-IgG. **F**, **G** ROCK1/AMPK activities in cells with the transfection of sh-circ_0057558 or sh-NC following FFA or control treatment. **H** Protein levels of ROCK1 and p-AMPK in cells with the transfection of sh-circ_0057558 or sh-NC following FFA or control treatment. **I** Quantifications of **H**. *p < 0.05, **p < 0.01, and ***p < 0.001.

To study the molecular mechanisms underlying the regulation of circ_0057558 on lipogenesis, we first examined the levels of miR-206 and ROCK1 in FFA-treated cells following circ_0057558 knockdown. Consistent with the aforementioned results, FFA treatment deceased miR-206 but increased ROCK1 level (Fig. 6A, B). Interestingly, knockdown circ_0057558 reversed those changes, recovering the level of miR-206 and decreasing ROCK1 (Fig. 6A, B). It has been shown that circRNAs can act as a miRNA sponge. Using bioinformatic analysis tool (Starbase, http://starbase.sysu.edu.cn/index.php), we found some complementary bindings sites between circ_0057558 and miR-206 (Fig. 6C). To directly examine the interaction, we used dual-luciferase reporter assay. miR-206 mimics significantly decreased the luciferase activity of circ_0057558-WT reporter but not the circ_0057558-MUT reporter wherein the predicted binding sites were mutated (Fig. 6D). To further confirm that interaction, we performed RNA immunoprecipitation. Immunoprecipitation with AGO2 antibody greatly pulled down both the circ_0057558 and miR-206 in liver cells compared with immunoprecipitation with normal IgG antibody (Fig. 6E). These results demonstrate that circ_0057558 directly binds with miR-206. We also measured how circ_0057558 regulated AMPK signaling and found that knockdown of circ_0057558 suppressed the elevation of ROCK1 level and activity induced by FFA treatment and recovered the p-AMPK level and AMPK activity (Fig. 6F–I). Taken together, we show that circ_0057558 inhibits AMPK signaling via targeting miR-206 to increase ROCK1 expression.

### Circ_0057558 promoted lipogenesis via targeting miR-206/ROCK1 axis

**Fig. 7:**
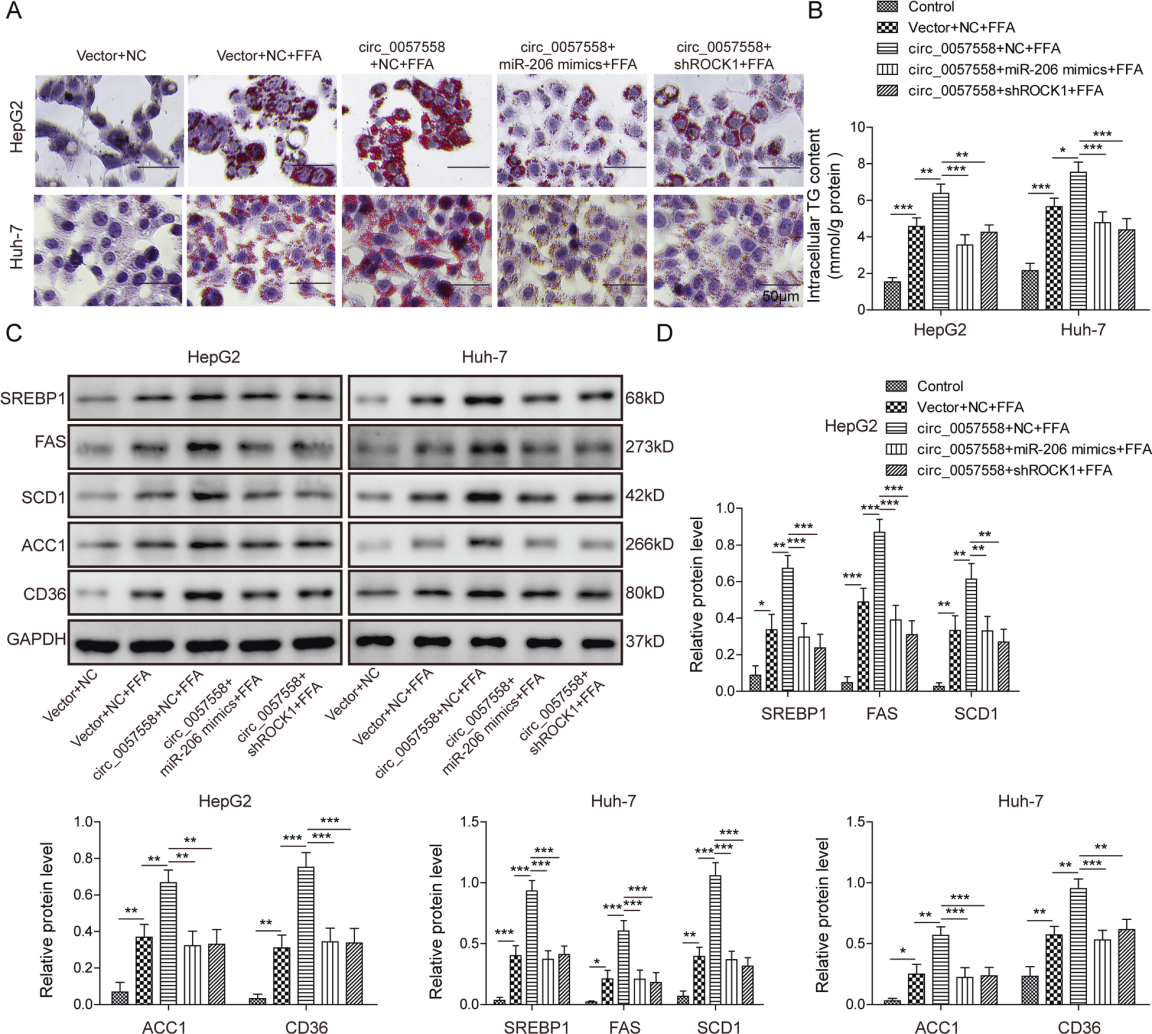
Circ_0057558 promoted lipogenesis via targeting miR-206/ROCK1 axis. **A** Representative oil-red o staining images of transfected circ_0057558 overexpressing vector cells following FFA or control treatment. **B** Secreted TG levels in transfected circ_0057558 overexpressing vector cells following FFA or control treatment. **C** Protein levels of lipogenesis-related proteins in transfected circ_0057558 overexpressing vector cells following FFA or control treatment. **D** Quantifications of **C**. *p < 0.05, **p < 0.01, and ***p < 0.001.

Next, we investigated how circ_0057558 regulated lipogenesis and we focused on its target miR-206. Consistent with the results above, overexpression of circ_0057558 enhanced the effects of FFA on lipogenesis, leading to more lipid deposition and higher levels of TG and lipogenesis-related proteins compared to FFA treatment alone (Fig. 7A–D). However, co-transfection of miR-206 mimics or sh-ROCK1 reversed the effects of circ_0057558 overexpression on lipogenesis, greatly suppressing the promotion (Fig. 7A–D). Therefore, these data show that circ_0057558 facilitates lipogenesis through miR-206/ROCK1 axis.

## Discussion

The prevalence of NAFLD is continuously on the rise and the current rising incidence of NAFLD raises a serious health concern to individuals and societies [1–3]. By understanding the molecular mechanisms underlying NAFLD development and progression, we can design novel therapeutic strategies to treat and prevent NAFLD. Here, we showed that circ_0057558 and miR-206 play a key role in the development of NAFLD. Circ_0057558 was increased while miR-206 was reduced in NAFLD. Knockdown of circ_0057558 and overexpression of miR-206 both could suppress the lipid accumulation and TG secretion in NAFLD. Mechanistically, we found that miR-206 directly targeted ROCK1 and activated AMPK signaling via ROCK1. circ_0057558 acted as a miR-206 sponge to inhibit AMPK signaling. Taken together, our study reveals an essential role of circ_0057558/miR-206/ROCK1/AMPK axis in NAFLD and thus this axis could serve as a molecular target for future therapy development.

miRNAs have crucial roles in many cellular processes, as well as in varieties of diseases, including cancers and metabolism-related disorders [25–28]. A great number of dysregulated miRNAs have been reported in NAFLD, such as miR-21, miR-140 [16, 29]. miR-206 has been shown to play key roles in development, cell proliferation, and differentiation [30, 31]. Also miR-206 is considered as a tumor suppressor via targeting oncogenes [32, 33]. Notably, miR-206 has been involved in metabolism as well. It has been reported that miR-206 can repress lipogenesis by targeting the liver X receptor α (LXRα) [34]. Further, miR-206 reduces lipid and glucose production in hepatocytes and the liver [18]. These previous studies implicate a potential role of miR-206 in NAFLD. In this study, we fully elucidated the function of miR-206 in NAFLD. We observed a lower level of miR-206 in NAFLD and overexpression of miR-206 suppressed the excess accumulation of lipid by inhibiting the expression of lipogenesis-related proteins like SREBP1, FAS, SCD1, ACC1, and CD36. Moreover, injection of miR-206 mimics could ameliorate NAFLD progression in vivo. Our study, together with other work, demonstrates a very important role of miR-206 in NAFLD.

CircRNAs are a class of covalently closed noncoding RNAs [21]. Since their discoveries, many circRNAs have been indicated to play key roles in cellular processes and diseases [22, 35]. Some of circRNAs have been implicated in NAFLD as well, such as circRNA_002581 and circRNA_29981 [36, 37]. Many circRNAs act as competing endogenous RNAs (ceRNAs) by sponging miRNAs [38]. For example, circRNA_002581 binds with miR-122 to disinhibit CPEB1 [39]. However, the studies of the role of circRNAs in NAFLD are very limited. Here, we identified a novel circRNA, circ_0057558, as a key regulator in NAFLD. The expression level of circ_0057558 has been shown correlated with TG level [23]. Here, we found a higher level of circ_0057558 in the NAFLD and knockdown of circ_0057558 significantly inhibited lipogenesis and TG secretion, indicating that circ_0057558 has a regulatory role in NAFLD development and progression. Mechanistically, we verified that circ_0057558 directly targeted miR-206 and regulated NAFLD through miR-206. It will be interesting to examine the functions of other circRNAs or other downstream miRNAs of circ_0057558 in NAFLD.

ROCK1 is a multifunctional protein that regulates many cellular processes, such as cell proliferation, adhesion, and motility [40]. In the past, dysregulated ROCK1 level has been reported in many metabolism-related disorders, including obesity, diabetes, and NAFLD [8, 41–43]. In NAFLD, previous studies show that the ROCK1 level is enhanced during NAFLD [7]. Increased ROCK1 functions to inhibit AMPK signaling which acts to restrain the activity of lipogenesis-related proteins such as SREBP1, SCD1 [44]. As a result, ROCK1 promotes the expression of those proteins and thus facilitates lipogenesis. Moreover, ROCK1/AMPK pathway is required for cannabinoid-induced lipogenesis in the liver [7]. However, the upstream regulators of ROCK1/AMPK signaling remain unknown. Here, we showed that miR-206 directly targeted ROCK1 and regulated NAFLD through ROCK1/AMPK signaling. In addition, circ_0057558 acts as a miR-206 sponge and promoted lipogenesis through ROCK1 as well. Therefore, our findings reveal a critical circ_0057558/miR-206/ROCK1/miR-206 axis that modulates de novo lipogenesis. Inhibition of ROCK1 activity has been shown to alleviate lipogenesis and thus can be potentially used for NAFLD therapy [7]. However, ROCK1 is a multifunctional kinase and ROCK1 inhibitor has broad effects. Our demonstration of circ_0057558 regulation of ROCK1 activity during NAFLD provides alternative ways to manipulate ROCK1 function specifically for NAFLD and minimize other side effects.

In summary, we provide evidence that circ_0057558 facilitates lipogenesis and contributes to NAFLD development by functioning as a sponge of miR-206, which directly targets ROCK1. The establishment of the crucial role of circ_0057558/miR-206/ROCK1/AMPK pathway in lipogenesis shed light on molecular mechanisms underlying NAFLD development and progression, and thus provides alternative avenues for NAFLD therapy.

## Materials and methods

### Nonalcoholic fatty liver disease (NAFLD) mouse model

All procedures related to animal husbandry and experiments have been approved by Animal Care and Use Committee of the Second Xiangya Hospital of Central South University. Male C57BL/6 J mice were obtained from SJA Laboratory Animal Co., Ltd (Hunan, China, *n* = 24) and kept in a standard 12 h/12 h light/dark cycle. The mice were randomly divided into four groups with six mice in each group. 8-week-old mice were fed with a high-fat (60 % kcal from fat, D12492, Research Diet, USA) diet for 12 weeks to induce NAFLD [45]. Control age-matched mice were fed with a regular diet. The investigator was blinded to the group allocation during the experiment. Mice will be excluded if they lose their appetite completely for 24 h or have a poor appetite (less than 50% of the normal amount) for 3 days. To measure the effects of miR-206 on NAFLD, miR-206 mimics [2 × 10^10^ plaque-forming units (pfu)) or mimics NC (2 × 10^10^ pfu) dissolved in PBS was injected into the mice through tail vein at the beginning of HFD (60% kcal from fat, D12492, Research Diet, USA) feeding. To harvest the liver tissues of the mice, mice were euthanized first and liver tissues were dissected out on the ice followed by snap-frozen in liquid nitrogen for subsequent biochemistry experiments or fixation in formalin for subsequent staining experiments.

### Cell culture and treatment

Two human liver cell lines (Huh-7 and HepG2) were used for the study and they were obtained from Chinese Academy of Sciences Cell Bank (Shanghai, China). Cells were cultured in the medium composed of Dulbecco’s modified Eagle medium (DMEM, Sigma-Aldrich, USA) plus 10% fetal bovine serum (FBS, Neuromics, USA) and 1% penicillin-streptomycin (Gibco, USA). All cells were grown in the standard cell incubator under standard conditions (5% CO_2_ and 37 °C). Cells were tested without contamination with mycoplasma. To induce free fatty acid (FFA) overloading, cells were grown up to 70~80% confluence and then treated with a mixture of long-chain FFA (oleic acid:palmitic acid = 2:1) at the concentration of 1 mM for 24 h.

### Sanger sequencing, actinomycin D and RNase R treatment

Circ_0057558 sequence was obtained using divergent primers sent to Sangon (Shanghai, China) for Sanger sequencing analysis. To detect the half-life of circ_0057558, 2 mg/mL Actinomycin D or dimethylsulfoxide (Sigma-Aldrich, St. Louis, MO, USA) as a negative control was added into the cell culture medium. For RNase R treatment, total RNA (2 μg) was incubated for 1 h at 37 °C with or without 3 U/μg of RNase R (Epicentre Technologies, USA). After treatment with Actinomycin D and RNase R, qRT-PCR was used to measure the expression levels of circ_0057558 and SLC39A10 mRNA.

### Fluorescence in situ hybridization (FISH) assay

Commercial FISH Kit (RiboBio, Guangzhou, China) was used for this assay. The culture medium was discarded followed by wash with PBS. 4% paraformaldehyde (PFA) was added to fix the cells by incubation for 13–15 min at room temperature. PFA was washed out by PBS and 0.5% Triton X-100 in PBS was used to permeabilize the cells for 3–5 min at room temperature. Fluorescence-labeled probes specific for circ_0057558 were added to incubate with cells overnight at 37 °C. The probes were washed out by PBS and stained cells were mounted on the slides by DAPI-containing mounting media (DAPI-Fluoromount-G, Thermo Fisher).

### Cell transfection

Circ_0057558 full length was cloned into the overexpression vector (pcDNA3.1). miR-206 mimics, inhibitor, ROCK1-shRNA, and circ_0057558-shRNA were synthesized and purchased from Genepharma (Shanghai, China). The transfection was performed by using the Lipofectamine 3000 reagent (Invitrogen, USA) following the guidance of the manufacturer. In brief, cells were cultured up to 70~80% confluence and then plasmid (1 μg) together lipofectamine 3000 (1 μL) was added into the medium. 48 h after transfection, transfected cells were collected for other experiments.

miR-206 inhibitor: 5’-CCACACACUUCCUUACAUUCCA-3’;

inhibitor NC: 5’-CAGUACUUUUGUGUAGUACAA-3’;

miR-205 mimics sense: 5’-UGGAAUGUAAGGAAGUGUGUGG-3’;

miR-205 mimics antisense: 5’-ACACACUUCCUUACAUUCCAUU-3’;

Mimics NC sense: 5’-UUCUCCGAACGUGUCACGUTT-3’;

Mimics NC antisense: 5’-ACGUGACACGUUCGGAGAATT-3’;

ROCK1-shRNA: 5’-GGTTAGAACAAGAAGTAAA-3’;

sh-NC: 5’-AATTGGGACGAAGAATAA-3’.

### Enzyme-linked immunosorbent assay (ELISA)

Triglyceride (TG) level was measured by the commercial ELISA kit (Nanjing, China) as the protocol described. The values obtained were normalized to total protein concentrations. Protein concentrations were detected using the BCA method.

### Oil-Red O staining

Oil-Red O staining was carried out by using the Oil-Red O staining kit (Abcam, USA) following the instructions of the manufacturer. For cells, briefly, the medium was discarded first and PBS was added to wash the cells. The cells were then fixed with 4% paraformaldehyde (PFA) at room temperature for 10–15 min. After the fixation, cells were stained with the Oil-Red O solution for 10–15 min at room temperature. The solution was then removed and water was added to wash off the solution. Hematoxylin was added to incubate with the stained cells for an additional 1–2 min and then water was used to wash cells again before imaging. For liver tissues, frozen sections were fixed in PFA. The sections were incubated with Oil-Red O solution for 10–15 min at room temperature as described above.

### Dual-luciferase report assay

cDNAs that contain the wild type (WT) sequences or mutated (MUT) binding sites of miR-206 in ROCK1 3’-UTR and circ_0057558 were cloned into downstream of the luciferase report vector (psiCHECK2). Phusion site-directed Mutagenesis kit (Thermo Fisher Scientific, MA, USA) was used to mutate the predicted binding sites as the protocol described. Human liver cells were cultured in 24-well culture plates until 70~80% confluence and then recombinant constructs were transfected into the liver cells using lipofectamine 3000 together with miR-206 mimics or NC. After 48 h, the cells were harvested in Reporter Lysis Buffer from the commercial kit (Promega, WI, USA) and the luciferase activities were measured.

### RNA immunoprecipitation (RIP) assay

Transfected cells (HepG2) were lysed with the lysis buffer (180 mM NaCl,50 mM Tris-HCl, 2 mM EDTA, 1% Triton X-100, 0.5% sodium deoxycholate) supplemented with RNase inhibitors and protease inhibitor cocktail (Sigma-Aldrich, USA) followed by centrifuge at 13,000 rpm for 12–15 min at 4 °C. The supernatant was saved to quantify the protein concentration using the commercial kit (Pierce BCA protein Assay kit, Thermo Fisher Scientific, MI, USA). Equal amount of the extracted protein (200 μg) was incubated with relevant antibodies (anti-AGO2, 1:800 dilution, ab186733; IgG was used as control, 1:1000) (Abcam, MA, USA) overnight at 4 °C and then pulled down by protein G Sepharose 4 Fast Flow (Sigma-Aldrich, MO, USA). Proteinase K (Sangon, Shanghai, China) was added to digest the beads for 1 h followed and elution was used for RNA purification by using Trizol reagent (Invitrogen, Missouri, USA). Quantitative RT-PCR was performed to examine the RNA yield. The primers were listed in the qRT-PCR section.

### ROCK1 and AMPK activity assay

The activities of ROCK1 and AMPK kinases were measured by the corresponding activity assay kits (ab211175, ab181422, Abcam, USA) as the manufacturer’s protocol described. Briefly, transfected cells following treatment were harvested and incubated with corresponding substrates for 1 h followed by antibody detection to measure substrate signaling.

### Hematoxylin and eosin (H&E) staining

H&E staining was carried out as the standard protocol described. Briefly, liver tissues were fixed in 10% formalin overnight at 4 °C first and subsequently embedded in paraffin. Paraffin sections (5 μm thick) were incubated with hematoxylin for 3 min first and then washed with water. Later, eosin was added to stain the slices for 30 s followed by dehydration with ethanol. Stained slices were mounted and then imaged with the microscope.

### Immunohistochemistry

Liver tissues were fixed in 10% formalin overnight at 4 °C and then embedded in paraffin followed by slicing into 5 μm thick sections. The liver slices were mounted on the glass and dried in the oven for 1 h at 60 °C. Slices were deparaffinized by washing with xylene and rehydrated through a graded concentration of alcohol. The tissue sections were washed with TBST and then blocked with 3% bovine serum albumin (BSA) for 1 h at room temperature followed by incubation with primary antibody (anti-ROCK1; 1: 1,000; PA5-22262, Invitrogen, USA) overnight at 4°C. The next day the primary antibody was removed and PBS was used to wash the slices. Secondary antibodies were added to incubate the slices for an additional 1 h at room temperature. Slices were washed again with PBS and then a commercial kit (Abcam, USA) was used to detect the signal following the guidance of the manufacturer. A light microscope was used to take images of stained slices.

### RNA extraction and RT-qPCR

Liver tissues or cultured cells were lysed in Trizol reagent (Invitrogen, Missouri, USA) supplemented with DNase I to extract total RNA as the manufacturer’s protocol described. Total RNA 1–2 μg from each sample used for reverse transcription to generate cDNAs using the standard kit (Invitrogen, MA, USA) and the cDNAs were subjected to PCR amplification (Invitrogen, MA USA). Relative expression levels of circ_0057558/mRNAs and miRNA were normalized to GAPDH mRNA or U6 RNA, respectively, as internal controls. The relative expression levels were calculated using the 2^-∆∆Ct^ method after normalization with reference control. The following primers were used:

circ_0057558 forward primer: 5’-CACACTTTGGATCTTTGCAGTCA-3’;

circ_0057558 reverse primer: 5’-GGAACCAAGATCACGCCTAGC-3’;

miR-206 forward primer: 5’-GTTATGGAATGTAAGGAAGTGTGTGG-3’;

miR-206 reverse primer: 5’-CCATCATGCTCTCGACCTGTC-3’;

ROCK1 forward primer: 5’-ACCTGTAACCCAAGGAGATGTG-3’;

ROCK1 reverse primer: 5’- CACAATTGGCAGGAAAGTGG-3’;

U6 forward primer: 5’-CTCGCTTCGGCAGCACA-3’;

U6 reverse primer: 5’-AACGCTTCACGAATTTGCGT-3’;

GAPDH forward primer: 5’-GAGTCAACGGATTTGGTCGTT-3’;

GAPDH reverse primer: 5’-TTGATTTTGGAGGGATCTCG-3’.

### Western blot

Liver tissues or cultured cells were lysed in the commercial RIPA buffer (Beyotime Institute of Biotechnology, Nantong, China) containing protease inhibitor cocktail followed by spinning down at 15,000 rpm for 12–15 min. The supernatant was saved to quantify the protein concentration using the commercial kit (Pierce BCA protein Assay kit, Thermo Fisher Scientific, MA, USA). Equal amounts of protein from each sample were loaded into the SDS-PAGE gels and separated through electrophoresis. The proteins in the gels were then transferred into the nitrocellulose membranes (Bio-Rad, CA, USA). The membranes were blocked in 3% BSA for 0.5 h at room temperature and then primary antibodies were added for overnight incubation at 4 °C. The next day, the membranes were washed with TBST and secondary antibodies were added for incubation for an additional 1 h at room temperature. Signals were detected using the commercial ECL kit (Sigma-Aldrich, MO, USA). The following primary antibodies were used: Anti-ROCK1 (1: 1000; ab97592, Abcam, USA); Anti-p-AMPK (1:500; 2535 S, Cell Signaling, USA). Anti-AMPK (1:1000; 2532, Cell Signaling, USA); Anti-SREBP1 (1:1000; ab28481, Abcam, USA); Anti-FAS (1:1000; 3180, Cell Signaling, USA); Anti-SCD1 (1:1000; ab236868, Abcam, USA); Anti-ACC1 (1:500; ab72046, Abcam, USA); Anti-CD36 (1:1000; ab252923, Abcam, USA); Anti-GAPDH (1:2000; ab9485, Abcam, USA).

### Statistical analysis

All experiments were performed with at least three times. The error bars indicated ± standard deviation (SD). All data were in a normal distribution, and variance was similar between the groups that are being statistically compared. Statistical analyses were analyzed in GraphPad Prism 7. Statistical significance was determined by using unpaired Student *t* test for two groups or one-way ANOVA when there are more than two groups. *p* < 0.05 was considered significant.

## Supplementary information

Supplementary Figure legends

Figure S1

Figure S2

Figure S3

## Data Availability

All data generated or analyzed during this study are included in this article. The datasets used and/or analyzed during the current study are available from the corresponding author on reasonable request.
