# Supplementary material for: Circ_0057558 promotes nonalcoholic fatty liver disease by regulating ROCK1/AMPK signaling through targeting miR-206
Source: Cell Death Dis. 2021 Aug 26;12(9):809. doi: 10.1038/s41419-021-04090-z (PMC8390503; doi:10.1038/s41419-021-04090-z)
Supplement: Supplementary file 1 — Supplementary Figure legends [file 41419_2021_4090_MOESM1_ESM.docx]

**Figure S1. Expression levels of circ_0057558, miR-206 and ROCK1 in transfected liver cells.** (A) Relative miR-206 levels in cells transfected with mimics NC, miR-206 mimics, inhibitor NC, or miR-206 inhibitor (B) Relative circ_0057558 levels in cells transfected with vector, circ_0057558, shNC, or sh-circ_0057558. (C) Relative ROCK1 mRNA levels in cells with the transfection of shNC or shROCK1. (D) Relative ROCK1 protein levels in cells with the transfection of shNC or shROCK1. **p*< 0.05, ***p*< 0.01, and ****p*<0.001.

**Figure S2 and Figure S3.** All original WB results with labeled molecular size
