# Supplementary figures and images for: Circ_0057558 promotes nonalcoholic fatty liver disease by regulating ROCK1/AMPK signaling through targeting miR-206
Source: Cell Death Dis. 2021 Aug 26;12(9):809. doi: 10.1038/s41419-021-04090-z (PMC8390503; doi:10.1038/s41419-021-04090-z)

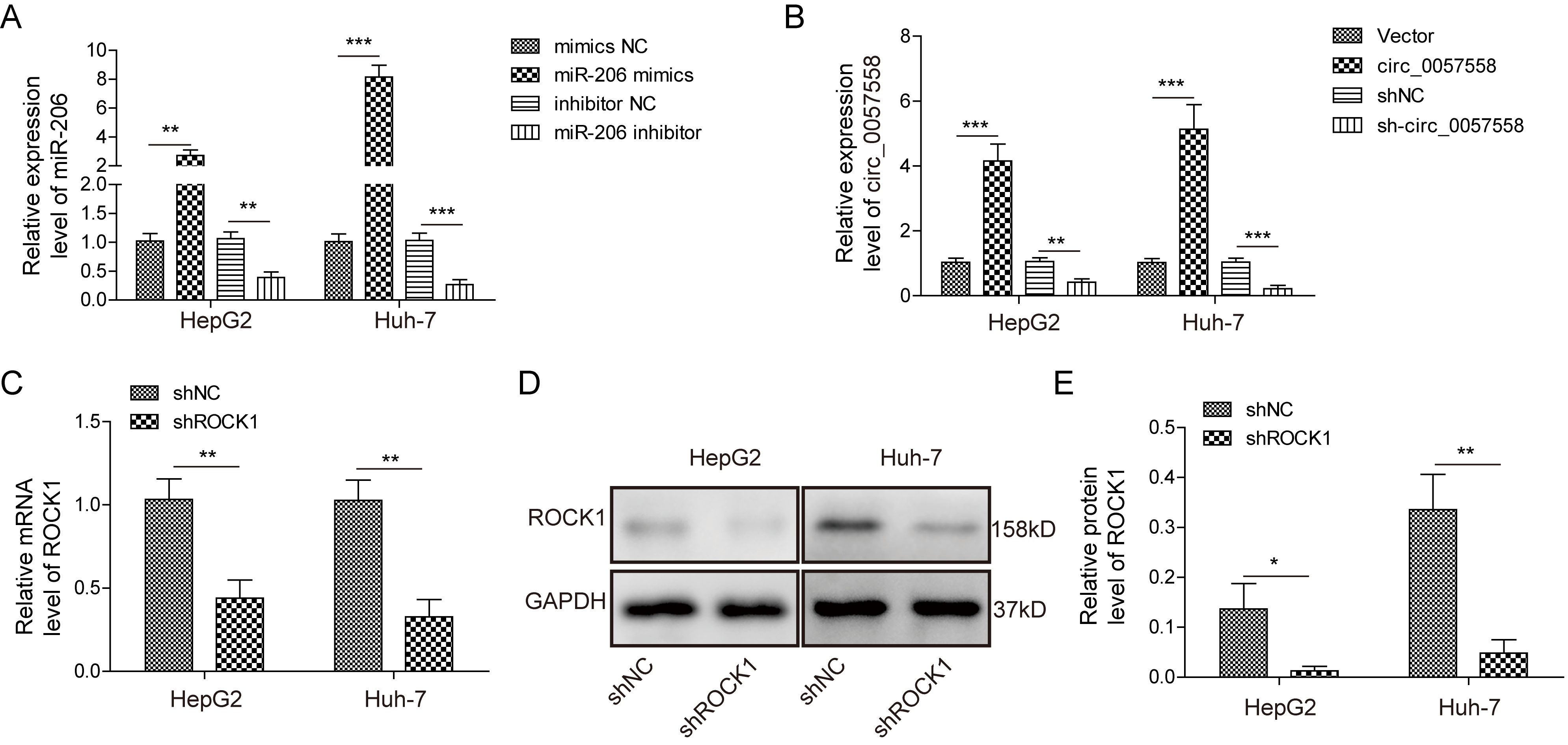

Supplement: Supplementary file 2 — Figure S1 [file 41419_2021_4090_MOESM2_ESM.jpg]

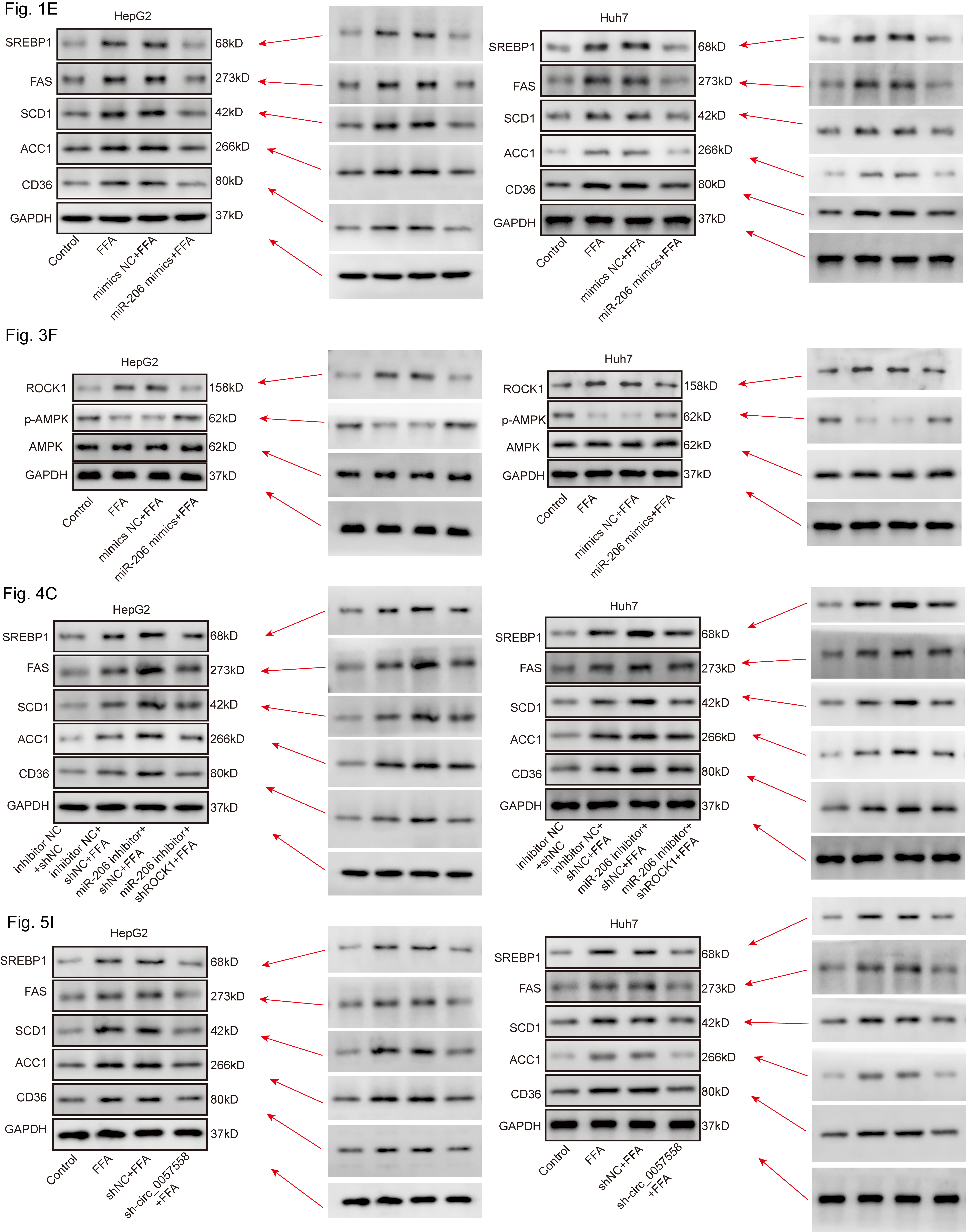

Supplement: Supplementary file 3 — Figure S2 [file 41419_2021_4090_MOESM3_ESM.jpg]

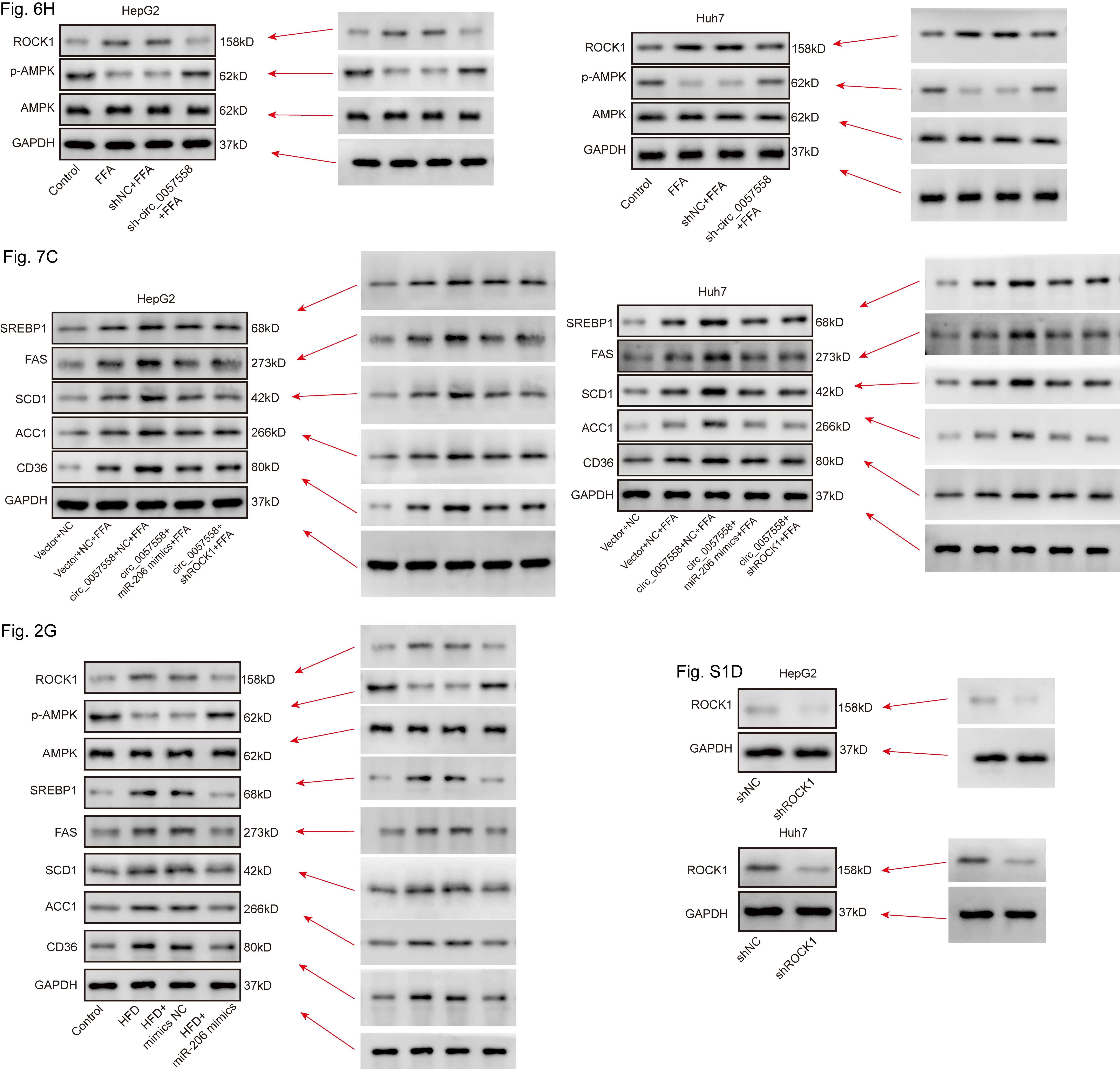

Supplement: Supplementary file 4 — Figure S3 [file 41419_2021_4090_MOESM4_ESM.jpg]
